# Construction of a nursing internship system through institutional collaboration: current status, challenges, and optimization pathways

**DOI:** 10.3389/fmed.2026.1867526

**Published:** 2026-09-07

**Authors:** Yijia Ren, Chunyan Li

**Affiliations:** School of Nursing, Hunan University of Chinese Medicine, Changsha, China

**Keywords:** clinical practice, internship, nursing education, preceptorship, talent training, university-hospital collaboration

## Abstract

Nursing internship serves as a pivotal link between nursing education and clinical practice, functioning as a key platform for cultivating applied nursing professionals. The university-hospital collaboration model integrates the resource advantages of both academic institutions and medical institutions, promoting the deep integration of theoretical knowledge and clinical skills, thereby significantly enhancing the practical abilities and professional competitiveness of nursing undergraduates. Although this model has achieved positive results in expanding practical opportunities, deepening resource sharing, and strengthening professional competence cultivation, it still faces prominent issues such as uneven mentor qualifications, inadequate pre-service training systems, incomplete internship management mechanisms, insufficient student rights protection, and weakened practical education functions. This paper proposes that standardized selection and training of mentors, the establishment of a comprehensive practical teaching system, enhanced protection of interns’ rights and safety, implementation of humanistic care and psychological support, and the creation of a university-hospital collaborative management mechanism should be adopted to standardize nursing education and precisely align with clinical needs. The optimized university-hospital collaboration model can effectively improve the quality of nursing talent cultivation, stabilize the nursing workforce, adapt to the demands of public health services, promote professionalization and high-quality development in nursing, and provide solid talent support for the implementation of the Healthy China strategy.

## 1 Introduction

Clinical internship is a core component of undergraduate nursing education, and a critical stage for the transformation of theoretical knowledge into clinical practice and the transition of professional skills into professional ethics. It plays an irreplaceable role in the cultivation of applied nursing talents (1, 2). Nursing undergraduate students entering real clinical scenarios can complete skill training, thinking shaping, and role adaptation under standardized practice and targeted guidance, forming a closed loop of “practice reflection relearning” ability improvement (3–5). At the same time, internship experience plays an important supporting role in building professional identity, establishing interpersonal networks, enhancing employment competitiveness, and positioning career directions. It is an important bridge for nursing students to transition from campus to clinical positions (6–10).

Against the backdrop of continuous improvement in the nursing education system, institutional cooperation has become the mainstream model for optimizing internship quality and enhancing educational effectiveness. This model focuses on the development of nursing students’ abilities, effectively connects classroom teaching with clinical needs through the integration of school and college resources, teaching collaboration, and quality management, and promotes the standardized and systematic development of practical teaching (11, 12). Relying on the real diagnosis and treatment environment of the hospital and the advantages of clinical faculty, combined with the support of universities in teaching design, process control, and scientific research cultivation, institutional cooperation can significantly improve the prominent problems of theoretical and practical disconnection, insufficient practical opportunities, and uneven teaching quality, enhance the clinical adaptability, learning initiative, and comprehensive practice ability of nursing students (13–15).

From the perspective of educational value, the cooperation model between universities has three meanings: individual development, educational improvement, and social service (16). For nursing students, expanding practical opportunities, sinking clinical resources, and providing standardized guidance can help strengthen their clinical competence; For nursing education, school hospital collaboration promotes innovation in teaching models and continuous improvement in quality; For the development of clinical institutions and industries, collaborative education can help optimize the talent supply structure and enhance the overall quality of the nursing team (17–20). Against the backdrop of the promotion of the Healthy China strategy, the professionalization transformation of nursing services, and the continuous upgrading of social health needs, high-quality clinical internships have become a key link in ensuring the supply of nursing talents, stabilizing the nursing team, and improving the level of medical services.

**Figure 1:**
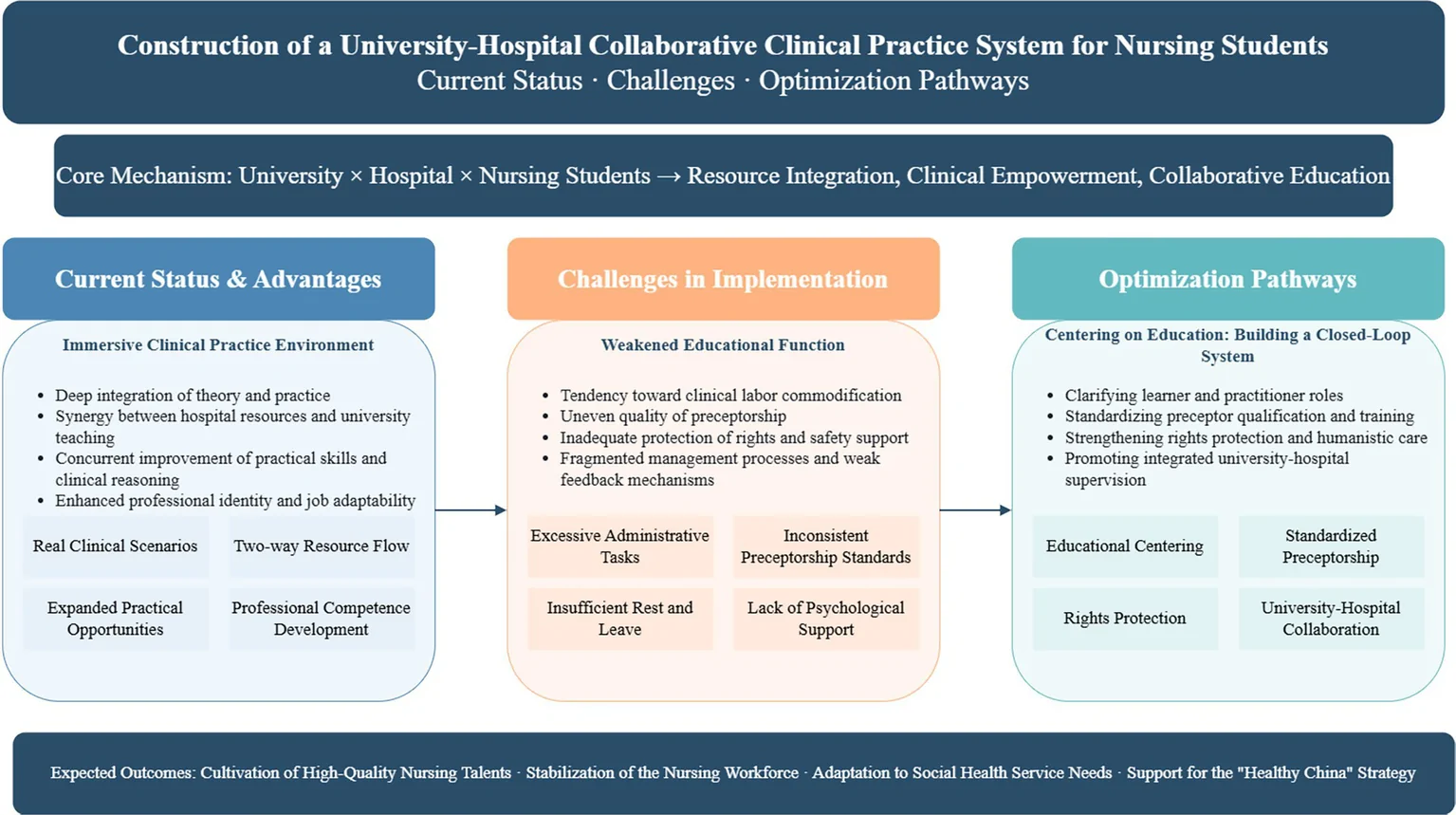
Flow chart.

However, the current undergraduate nursing internships in China still face multiple structural challenges in practice, including a clear tendency towards employment, uneven teaching quality, insufficient protection of student rights, and fragmented training and management, which seriously restrict the effectiveness of education and industry retention rate (21–24). This research integrates institutional practical practice and literature review for evidence synthesis. It analyzes the practical problems of the local nursing internship model based on the author’s institutional teaching practice and complements theoretical support through sorting out relevant domestic and foreign literatures. Based on this, this article starts from the current situation and intrinsic value of internships, systematically sorts out the current practical difficulties, and proposes targeted optimization paths, providing theoretical references and practical perspectives for building a standardized, scientific, and humanized nursing undergraduate internship system (Figure 1).

## 2 Current status and advantages

In the context of the professionalization of nursing, clinical internship is a key carrier for achieving the transformation of theoretical knowledge into clinical practice and the transition of professional skills into professional abilities. At present, nursing internships generally adopt a collaborative organizational model between universities and internship hospitals. Through the integration and collaborative linkage of resources between universities and internship hospitals, a practical platform that is close to real diagnosis and treatment scenarios is provided for nursing students, significantly improving the authenticity and standardization of practical teaching (25). Relying on the complete diagnosis and treatment process, disease resources, and work scenarios of the hospital, nursing students can internalize professional knowledge, train operational skills, develop clinical thinking, and adapt to professional roles through immersive practice, effectively bridging the gap between classroom teaching and clinical practice. Under the framework of institutional cooperation, schools and hospitals achieve a two-way flow of faculty, teaching, management, and service resources. Clinical experts from hospitals participate in practical guidance and skill teaching, integrating cutting-edge clinical concepts, standardized operating standards, and real cases into the teaching process; Universities are responsible for designing internship programs, quality monitoring, and process evaluation, promoting the standardized and systematic development of practical teaching. At the same time, standardized clinical internships can systematically enhance the professional ethics, communication skills, sense of responsibility, and humanistic care abilities of nursing students, and strengthen their sense of professional identity and job adaptability. Overall, the school cooperation model provides stable organizational support, rich practical scenarios, and comprehensive teaching support for nursing internships, playing an irreplaceable role in promoting the comprehensive development of nursing students, enhancing employment competitiveness, and ensuring the supply of clinical nursing talents.

## 3 Challenges in implementation

There are still deep-seated structural contradictions in the current undergraduate nursing internship system in China, and the core function of practical education has been significantly weakened (26). Firstly, there is a significant trend towards employment oriented internships, which dilutes the value of teaching. Internship institutions generally position interns as auxiliary labor force, arranging a large number of repetitive and low technical basic transactional work. There is a serious lack of opportunities for core clinical operation training and thinking cultivation, and nursing students are mostly in a state of “passive labor and shallow participation”; In addition, due to the lack of corresponding guarantees and incentives, nursing students have a low sense of practical gain, and their professional enthusiasm and willingness to work continue to decline (27, 28). Secondly, the teaching system is not perfect, and the quality of teaching varies greatly. The admission, training, assessment, and incentive mechanisms for mentoring are not yet sound, and there are significant differences in the teaching awareness, guidance ability, and professional ethics of mentoring personnel; The problems of unreasonable teaching allocation, discontinuous guidance, and insufficient teacher-student interaction are prominent, making it difficult to form a stable and efficient guidance relationship, which directly restricts the effectiveness of practical teaching (29). Thirdly, the protection of students’ rights and interests is weak, and there is insufficient support for professional safety and humanities. The basic rights and interests of interns, such as the right to rest and vacation, are difficult to effectively implement. The leave process is cumbersome, departmental responsibilities are unclear, and there is a widespread phenomenon of shirking responsibilities. Some institutions have problems such as forced overtime and overloaded work; The basic guarantee conditions for internship are not perfect, the prevention and control of professional risks and safety protection are not in place, the mental health support system is lacking, and the sense of security and belonging of nursing students is insufficient (30, 31). Fourthly, teaching and management are fragmented, resulting in low educational effectiveness. Pre job training is formalized and fragmented in content, with insufficient practical training and scenario based teaching, making it difficult to form an embedded training model that runs through the entire internship process; The lack of a sound mechanism for collaborative management between schools and colleges, coupled with weak abilities in process monitoring, dynamic feedback, and emergency response, further reduces the overall operational efficiency and quality of education of the internship system (32).

## 4 Optimization pathways

One is to return to the education oriented approach and correct the tendency towards internship employment. Clarify the subject positioning of “learner+practitioner” for nursing students, reasonably define the boundary between teaching tasks and transactional work, construct a stepped clinical ability training mechanism, gradually improve the operational participation and thinking training intensity of nursing students under safety supervision, and make internships return to the core goals of ability cultivation and career shaping (33, 34). The second is to standardize teaching management and build a high-quality practical teaching system. Improve the admission standards for teaching, systematize training systems, and diversify assessment and incentive mechanisms, promote fixed and responsible teaching models, strengthen the teaching ability, sense of responsibility, and humanistic literacy of teaching personnel, and enhance the continuity, standardization, and professionalism of guidance. The third is to strengthen the protection of rights and interests, improve the security prevention and control and humanistic support system. Implement a reasonable schedule and vacation system, simplify management processes, clarify division of responsibilities, and eliminate illegal overtime and overloaded employment; Establish a sound mechanism for preventing and controlling professional risks and occupational safety protection, improve basic guarantee conditions, build a normalized mental health service and humanistic care system, and enhance the sense of security and professional belonging of nursing students during internships (35). The fourth is to deepen the collaboration between schools and colleges, and promote integrated internship management. Establish a fully embedded practical teaching mode, integrating skill training, standardized explanation, and problem feedback into the daily internship process, achieving simultaneous practice, learning, and improvement; Improve the joint supervision between schools and colleges, information technology process monitoring, and rapid response mechanism, promote the transformation of internship management towards refinement, scientificity, and humanization, and comprehensively enhance the quality of education and sustainable development ability of nursing undergraduate internships (36, 37).

## 5 Implementation considerations

The operation of the university-hospital collaborative management mechanism is safeguarded by a clear governance framework, smooth communication channels, and comprehensive accountability measures. In terms of governance structure, a collaborative management system is established, comprising the university, hospital nursing department, departmental chief instructors, fixed instructors, and interns, with clearly defined responsibilities and authorities for each party. Regarding communication channels, a mechanism is implemented where counselors organize weekly online safety check-ins to dynamically monitor students’ on-the-job status and safety conditions, combined with the university’s random clinical supervision, monthly specialized training, and biweekly joint meetings between the university and hospital. As for accountability measures, the nursing department’s specialized supervision team conducts teaching evaluations and rights supervision to ensure operational standards and safety management are implemented. During implementation, supporting resources such as faculty, training funds, and an information management platform are required to promote the collaborative participation of multiple stakeholders—universities, hospitals, instructor teams, and interns. The effectiveness of optimization is systematically measured in practice through a multi-dimensional mixed assessment system, combining formative and summative evaluations: quantitative dimensions gather data through monthly operational assessments, mid-term comprehensive evaluations, final occupational competency scales, and tripartite satisfaction surveys; qualitative dimensions explore deeper issues and improvement directions through focus group interviews and in-depth interviews with interns, instructors, and nursing managers. The assessment results will be used to dynamically adjust training content, optimize instructor arrangements, and refine management mechanisms, ultimately achieving continuous iteration and quality improvement in the nursing internship system (38–42).

## 6 Conclusion

As a key bridge connecting campus education and clinical practice, nursing undergraduate clinical internships play an important role in enhancing nursing students’ clinical abilities, professional qualities, and employment competitiveness. The current nursing internship system still faces prominent challenges such as weakened emphasis on education, obvious tendency towards employment, uneven quality of teaching, insufficient protection of student rights, and fragmented management of schools and institutions, which directly affect the quality of nursing talent cultivation and industry retention rate.

In the future, it is necessary to focus on returning to the essence of education, strengthening collaboration between schools and colleges, standardizing process management, and safeguarding students’ rights and interests. By clarifying the dual positioning of “learner+practitioner” in nursing, improving the access and training system for teaching, establishing a sound mechanism for rights security and humanistic support, and building a fully embedded integrated management model, we can promote the transformation of nursing internships from “transactional employment” to “systematic education.”

## Data Availability

The original contributions presented in the study are included in the article/supplementary material, further inquiries can be directed to the corresponding author/s.

## References

[1] Hobenu KA Adefuye AO Naab F Nyoni CN. Strategies to enhance clinical teaching and learning in undergraduate nursing education: a scoping review. PLoS One. (2025) 20:e0305789. doi: 10.1371/journal.pone.0305789, 40493639 PMC12151355

[2] Wang L He LH Wan JH. Implementation of a guideline-based clinical practice teaching model to enhance learning and thinking abilities in undergraduate nursing students. BMC Nurs. (2025) 24:572. doi: 10.1186/s12912-025-03183-w, 40399879 PMC12093838

[3] Munangatire T McInerney P. A phenomenographic study exploring the conceptions of stakeholders on their teaching and learning roles in nursing education. BMC Med Educ. (2022) 22:404. doi: 10.1186/s12909-022-03392-w, 35619092 PMC9134698

[4] Pereira F Lehmann-Wellig B Verloo H. Enhancing beliefs and implementation of evidence-based practice among undergraduate nurses using a multi-component educational programme: a pre-post study. BMC Med Educ. (2025) 25:531. doi: 10.1186/s12909-025-07121-x, 40229643 PMC11998436

[5] Altinbas BC Çalık KY Erdöl EK Kırkbir İB Güner SG Tezel M . The effect of simulation-based laboratory training on undergraduate nursing students' clinical skill, satisfaction, and self-confidence. BMC Nurs. (2025) 24:1322. doi: 10.1186/s12912-025-04004-w, 41131550 PMC12551180

[6] Ling DL Ho KY Pan JD Zhang C Xu WT Lin KL . Implementation of a practice-based reflection training programme in undergraduate nursing interns for reflective practice competency and humanistic caring ability: a quasi-experimental study. Asian Nurs Res. (2026) 20:48–55. doi: 10.1016/j.anr.2025.09.005, 41224196

[7] Jia X Du J Xu T Yin X Zhang Q Bao X . Investigating the status and influencing factors of professional identity formation among Chinese medical interns: a mixed methods study. BMC Med Educ. (2025) 25:676. doi: 10.1186/s12909-025-07199-3, 40340896 PMC12063465

[8] Wei M Zhang Y Zhu F Zhai D Zhu C Zhao Y. From motivation to meaning in medical humanities: a mixed-methods study of Chinese medical students' value internalization. BMC Med Educ. (2026) 26:188. doi: 10.1186/s12909-025-08504-w, 41491187 PMC12870167

[9] Olive P Maxton F Bell CA Bench S Tinkler L Jones S . Clinical academic research internships: what works for nurses and the wider nursing, midwifery and allied health professional workforce. J Clin Nurs. (2022) 31:318–28. doi: 10.1111/jocn.15611, 33368730

[10] Abdalla ME Taha MH Onchonga D Nour N Kelly D Harney S . Exploring strategies, programs, and influencing factors for integrating social accountability into undergraduate medical education: a scoping review. BMC Med Educ. (2024) 24:1107. doi: 10.1186/s12909-024-06072-z, 39375698 PMC11460141

[11] Tang Y Linyue T Chen X Chen H. Construction and practice of a certification system for clinical nursing preceptors: a "license to teach" approach in China. BMC Med Educ. (2026) 26:606. doi: 10.1186/s12909-026-08953-x PMC13077961 41787491

[12] Yusefi AR Dehghani Z Nahardani SZ Ramezani G Keshavarzi MH. An analysis of facilitating and hindering factors in effective evidence-based medical education: a study within the context of medical education in Iran. BMC Med Educ. (2025) 25:1588. doi: 10.1186/s12909-025-08187-3, 41225471 PMC12613663

[13] Fessele KL Jefferson UT Clayton-Jones DL Jeffery AD LeBaron V Edward J . Cultivating an optimal environment for nursing innovation. Nurs Outlook. (2026) 73:102664. doi: 10.1016/j.outlook.2025.102664, 41771633

[14] Sebire NJ Adams A Arpiainen L Celi L Charlesworth A Gorgens M . The future hospital in global health systems: the future hospital as an entity. Int J Health Plann Manag. (2025) 40:730–40. doi: 10.1002/hpm.3893, 39748156 PMC12045724

[15] Huang P Miao W Wang R Yang F Li X Shen N. Innovative multimodal educational strategies: assessing the impact of integrative teaching methods on standardized neurology resident training. BMC Med Educ. (2025) 25:1032. doi: 10.1186/s12909-025-07507-x, 40640779 PMC12247243

[16] Wu L Chen Y Xue M Zhu W Wang W. The effect of social support on learning engagement among Chinese nursing interns: the mediating role of self-efficacy. BMC Nurs. (2025) 24:955. doi: 10.1186/s12912-025-03615-7, 40691600 PMC12278551

[17] Luo Y Chen Y Feng M. The perception of career planning and development among senior clinical nurses: a qualitative analysis. Int Nurs Rev. (2025) 72:e70050. doi: 10.1111/inr.70050, 40583117

[18] Yang H Chen Y Peng X. An ideal portrait of the professional competence of clinical research nurses: a qualitative study. Asia Pac J Oncol Nurs. (2025) 12:100682. doi: 10.1016/j.apjon.2025.100682, 40201532 PMC11976228

[19] Saleh MSM Ata AA Abd-Elhamid ZN Eltahan AA Dailah HG Elsabahy HE. Building nursing leaders: the influence of entrepreneurial leadership program on nurse interns' innovation and clinical performance. BMC Nurs. (2025) 24:501. doi: 10.1186/s12912-025-03100-1, 40340834 PMC12063326

[20] Ahmedy F Yin KN Sybil Shah S Darsin Singh SK Amat NAF Francis FA . Collaborative capacity building for strengthening rehabilitation services in Sabah, Malaysia: a partnership between university hospital and state health department. J Rehabil Med. (2025) 57:jrm40166. doi: 10.2340/jrm.v57.40166, 39907216 PMC11812271

[21] Xu Y Zhang W Wang J Guo Z Ma W. The effects of clinical learning environment and career adaptability on resilience: a mediating analysis based on a survey of nursing interns. J Adv Nurs. (2026) 82:2903–12. doi: 10.1111/jan.16144, 38468419

[22] Aliafsari Mamaghani E Rahmani A Hassankhani H Zamanzadeh V Campbell S Fast O . Experiences of Iranian nursing students regarding their clinical learning environment. Asian Nurs Res. (2018) 12:216–22. doi: 10.1016/j.anr.2018.08.005, 30165246

[23] Shen H Cui N Guo C Ma A Bian J. Night shifts and sleep among Chinese nursing students on internship. Occup Med (Lond). (2025) 75:518–25. doi: 10.1093/occmed/kqaf084, 40901710

[24] Chen S Liu W Li Y Zhang P Ye H Feng X . Transition shock among nursing interns: roles of achievement motivation and clinical practice environment-a cross-sectional study. Nurs Open. (2025) 12:e70297. doi: 10.1002/nop2.70297, 40911465 PMC12412515

[25] Shen M Feng Z Shen Y Shi Z. Enhancing undergraduate nursing informatics literacy through design-based learning: a mixed-methods participatory action research study. BMC Nurs. (2025) 24:1268. doi: 10.1186/s12912-025-03927-8, 41088142 PMC12523023

[26] Zhang S Zhang X Zhang L Zhang Q Liu H Li L . Burnout in transition: a qualitative study of nursing interns' experiences and implications for clinical management. J Nurs Manag. (2026) 2026:6694491. doi: 10.1155/jonm/6694491, 41510017 PMC12776595

[27] Lim S Xin Min L Chan CJW Dong Y Mikkonen K Zhou W. Peer mentoring programs for nursing students: a mixed methods systematic review. Nurse Educ Today. (2022) 119:105577. doi: 10.1016/j.nedt.2022.105577, 36179425

[28] Salameh B Abdallah J Malak MZ Shehadeh A Hammad B. Alarm fatigue and its association with perceived stress, resilience, and coping behaviors among Palestinian nursing students during clinical internship in critical care units: a cross-sectional study. BMC Psychol. (2025) 13:486. doi: 10.1186/s40359-025-02809-7, 40341074 PMC12063433

[29] Zhu Y Zhu J. Nursing students' competence and perceived mentor assessment literacy: the mediating role of self-efficacy and gender bias. BMC Nurs. (2025) 24:1243. doi: 10.1186/s12912-025-03862-8, 41057885 PMC12506267

[30] Negm LMMA Mersal FA Fawzy MS Rajennal AT Alanazi RS Alanazi LO. Challenges of nursing students during clinical training: a nursing perspective. AIMS Public Health. (2024) 11:379–98. doi: 10.3934/publichealth.2024019, 39027388 PMC11252586

[31] Dong S Shen X Zhao T Zeng R Chen M. Workplace violence, psychopathological symptoms, and deviant workplace behavior among nursing interns in China: a network analysis. BMC Nurs. (2025) 24:129. doi: 10.1186/s12912-025-02771-0, 39905417 PMC11796222

[32] Panda S Dash M John J Rath K Debata A Swain D . Challenges faced by student nurses and midwives in clinical learning environment - a systematic review and meta-synthesis. Nurse Educ Today. (2021) 101:104875. doi: 10.1016/j.nedt.2021.104875, 33774528

[33] Yao Q Luo J Ni Y He C Huang C Xu T . The impact of clinical learning environment on nursing interns' professional identity: the mediating role of belongingness. Nurse Educ Today. (2026) 160:106974. doi: 10.1016/j.nedt.2026.106974, 41534298

[34] Mohmand S Monteiro S Solomonian L. How are medical institutions supporting the well-being of undergraduate students? A scoping review. Med Educ Online. (2022) 27:2133986. doi: 10.1080/10872981.2022.2133986, 36268575 PMC9590426

[35] Squire D Gonzalez L Shayan C. Enhancing sense of belonging in nursing student clinical placements to advance learning and identity development. J Professional Nur. (2024) 51:109–14. doi: 10.1016/j.profnurs.2024.01.007, 38614668

[36] Tan M Liu H Xiang Y Xiong L Zou L Fang L. Practical research on the cultivation of inter-disciplinary undergraduate nursing talents in the context of "big health". BMC Med Educ. (2025) 25:717. doi: 10.1186/s12909-025-07075-0, 40382607 PMC12084930

[37] Zou Y Wang L Zheng D Li H Sun R Zhang Z. Exploration of undergraduate nursing students' perspectives on conducting specialized nursing education during internship: a phenomenological research study. Nurse Educ Pract. (2024) 80:104103. doi: 10.1016/j.nepr.2024.104103, 39216263

[38] Heydarikhayat N Ghanbarzehi N Sabagh K. Strategies to prevent medical errors by nursing interns: a qualitative content analysis. BMC Nurs. (2024) 23:48. doi: 10.1186/s12912-024-01726-1, 38233901 PMC10792785

[39] El-Sayed AAI Abdelaliem SMF. Application of Kano model for optimizing the training system among nursing internship students: a mixed-method Egyptian study. BMC Nurs. (2023) 22:316. doi: 10.1186/s12912-023-01485-5, 37710268 PMC10500916

[40] Shahzeydi A Farzi S Tarrahi MJ Sabouhi F Babaei S Yazdannik A. The effect of the clinical supervision model on nursing internship students' nursing process-based performance: an experimental study. BMC Nurs. (2024) 23:166. doi: 10.1186/s12912-024-01840-0, 38459482 PMC10921759

[41] Dokoohaki R Rambod M Pasyar N Parviniannasab AM Shaygan M Kalyani MN . Comparison of professional competency and anxiety of nursing students trained based on two internship models: a comparative study. BMC Med Educ. (2024) 24:968. doi: 10.1186/s12909-024-05956-4, 39232798 PMC11376021

[42] Allan JD Aldebron J. A systematic assessment of strategies to address the nursing faculty shortage, U.S. Nurs Outlook. (2008) 56:286–97. doi: 10.1016/j.outlook.2008.09.006, 19041450

